# Prevalence, awareness, treatment and control of diabetes among Iranian population: results of four national cross-sectional STEPwise approach to surveillance surveys

**DOI:** 10.1186/s12889-022-13627-6

**Published:** 2022-06-18

**Authors:** Rahmkhoda Khodakarami, Zhaleh Abdi, Elham Ahmadnezhad, Ali Sheidaei, Mohsen Asadi-Lari

**Affiliations:** 1 Department of Epidemiology, School of Public Health, Iran University of Medical Sciences (IUMS), Tehran, Iran; 2 National Institute of Health Research (NIHR), Tehran University of Medical Sciences, Tehran (TUMS), No 70, Bozorgmehr St. Vesale Shirazi St., Tehran, Iran; 3 Department of Epidemiology and Biostatistics, School of Public Health, Tehran University of Medical Sciences, Tehran, Iran; 4 Oncopathology Research Centre, Iran University of Medical Sciences (IUMS), Tehran, Iran

**Keywords:** Diabetes, Awareness, Prevalence, Treatment, Non-communicable diseases, Effective coverage, Universal health coverage

## Abstract

**Background:**

Diabetes as a leading cause of death imposes a heavy burden on health systems worldwide. This study investigated the trends in prevalence, awareness, treatment and control of diabetes among Iranian population aged 25 to 65 years over 12 years (2004-2016).

**Methods:**

Secondary data analysis was performed using data from a national population-based survey, STEPwise approach to surveillance (STEPS) for non-communicable diseases (NCDs) in four rounds (2004, 2007, 2011, 2016). The sample sizes were 89,404, 29,991, 12,103 and 30,541 individuals, respectively across the country in both rural and urban areas. Data were analyzed using descriptive statistics and a logistic regression model with odds ratio at a significance level of less than 5% with no adjustment for age and sex. Logistic regression was used to identify socio-demographic factors associated with the levels of awareness, treatment and control of diabetes mellitus.

**Results:**

The prevalence of diabetes in four rounds was 8.4, 9, 11.1 and 13.2%, respectively. Among people with diabetes, 53.5, 65.6, 70.5 and 82.2% were aware of their condition and 35.9, 42, 46 and 39.6% were treated for this condition, respectively. In four rounds of study, 14.5, 20.8, 20.4 and 18.5% of all diabetic patients had adequate glycemic control, respectively. In the multivariable logistic regression analysis, there was a significant relationship between female gender, age over 40, living in the urban area, being in the third wealth quintile and having health insurance with diabetes prevalence. Female participants were more likely to be aware of the disease. Older participants were more likely to receive treatment and had adequate glycemic control.

**Conclusion:**

The prevalence of diabetes in Iran has been increasing and despite the great awareness of the disease, receiving treatment and effective control of the disease are suboptimal. While several national policies to improve diabetes screening and care have been passed in recent years, it seems large gaps remain in disease detection and treatment. It is suggested that more attention be paid to the treatment and control of diabetes by NCDs national policies to prevent the growing burden associated with the disease.

## Background

Non-communicable diseases (NCDs), including diabetes, as one of the leading causes of death worldwide, are now one of the greatest challenges of the twenty-first century [1]. Diabetes is one of the top 10 causes of death globally. Together with cardiovascular diseases, cancer and respiratory disease, these conditions account for over 80% of premature NCDs deaths [2]. Although the incidence of diabetes has decreased in some developed countries, the prevalence of this disease is increasing in both developing and developed countries [3]. According to the prediction of the International Diabetes Federation, the global diabetes prevalence is estimated to be 9.3% (463 million people) in 2019, which will reach 10.2% in 2030 and 10.9% in 2045 [4]. The Middle East and North Africa (MENA) region, where Iran located there, has the highest prevalence of diabetes in the world. It was reported at 12.2% in 2019, which is expected to reach 13.3% in 2030 and 13.9% in 2045. The region is expected to witness a 96% increase in diabetes prevalence between 2019 and 2045. Further, 44.7% of individuals with diabetes in the MENA region are unaware of their condition [4]. World Health Organization (WHO), therefore, has focused on diabetes as a major global health concern in view of the enormous worldwide epidemic of this disease, perhaps the most important non-communicable global disease fostered by an unhealthy modern lifestyle [5].

According to a report published by the WHO in 2018, at least 10% of Iranians over the age of 18 have elevated blood glucose, which is higher than the estimated prevalence of raised blood glucose worldwide.  As estimated in the report, NCDs accounted for 82% of all deaths nationwide in 2016, with diabetes and cardiovascular disease directly responsible for at least 47% of total deaths [6]. Almost half of all deaths attributable to high blood glucose occur before the age of 70 years [7].

Diabetes and its associated complications have been recognized as a challenge to achieving the Sustainable Development Goals (SDGs) and Universal Health Coverage (UHC) worldwide. Emerging NCDs burden urged United Nations to call for one third reduction by 2025 in premature mortality from NCDs through prevention and treatment through SDG 3.4 [8]. In the context of UHC, NCDs have already received global attention and are given high priority [9]. Management and treatment of diseases such as diabetes is recognized as one of the main indicators to assess health system performance in the path towards UHC [10]. WHO has recommended the inclusion of management of diabetes as an indicator to monitor UHC for coverage of essential health services [11]. Achieving UHC has been a top priority in the Islamic Republic of Iran for over a decade. The most recent health sector reform—the Health Transformation Plan (HTP)— launched in 2014 by the Ministry of Health and Medical Education (MoHME), to provide UHC, including access to NCDs prevention and control services [12]. Given the increasing growth of NCDs burden in Iran, a dedicated national action plan for NCDs’ prevention and control was established under HTP. The action plan adapted WHO’s PEN (package of essential NCDs’ interventions for primary health care (PHC) in low-resource settings), so called IraPEN 2015-2025 strategy aiming at strengthen screening and primary care for NCDs including diabetes and hypertension as well as mental health services within PHC network in Iran [13]. Considering the huge burden of diabetes in Iran, this study aimed to estimate trends in prevalence, awareness, treatment, and control of diabetes using a 12-years period (2004-2016) nationwide population-based survey data.

### Delivering diabetes services in Iran health system

In Iran’s health system, services are provided at three primary, secondary and tertiary levels. Only preventive and consultant services are delivered at primary health care, which is free of charge for patients. Services related to diabetes delivered at the primary care level are visits by GPs, screening, basic medicine (e.g. Glibenclamide and metphormnie), basic technologies and procedures (e.g. blood glucose measurement, oral glucose tolerance test, urine strips for glucose and ketone measurement), and nutrition consultancy services. To receive other therapeutic services including visits of specialist physicians or more sophisticated laboratory tests, patients refer to secondary and tertiary healthcare providers at both public and private sector, which are not free of charge. At these levels, a significant part of treatment costs or medicine prices is paid directly by the patients and health insurance funds pay the rest. Factors associated with service delivery such as poor referral and follow up system, lack of workforce, medicine and facilities (e.g. medical laboratories) in PHC centers, and lack of integrated diabetes services, making diabetic patients who need different services spend additional time, resources and energy accessing care at separate clinics, were reported as the main weaknesses of the diabetes management in the public facilities of Iran [14].

## Methods

### Study objectives

The study objectives were:

1. To investigate trends of prevalence, awareness, treatment, and control of diabetes among Iranians aged 25-65 over 12 years.
2. To identify factors associated with prevalence, awareness, and control of diabetes among Iranians aged 25-65 over 12 years.

### Data source

This is a secondary data analysis using primary data from a population-based survey, WHO STEPwise approach to surveillance (STEPS) of risk factors for NCDs. STEPS is a WHO-developed, standardized framework for countries to monitor the main NCDs risk factors through questionnaire assessment and physical and biochemical measurements. STEPS surveys are implemented at the country level as national household surveys using trained interviewers who undertake face-to-face interviews at household level with selected survey respondents of behavioral risk factors and physical measurements such as blood pressure and height and weight measurements (steps 1 and 2). Step-3 biochemical assessments for blood glucose, blood lipids, and urinary sodium usually take place at a local clinic or health center [15].

STEPS survey has been implemented for seven rounds in Iran in years of 2004, 2006, 2007, 2008, 2009, 2011 and 2016 [16]. The first two steps were implemented in all years and the third step, which collects data on biochemical measures including blood glucose, was performed in four years (2004, 2007, 2011, 2016). All Iranians aged 18 years who were living in Iran at the time of data collection were eligible for inclusion in the STEPS surveys but taking blood samples were limited to individuals who were 25 years and above. In this study, we included those studies in which laboratory measurements carried out through step 3 on a subsample population.

The detailed methodology of the STEPS surveys performed in Iran has been published elsewhere [17, 18]. Despite slight differences in the sampling designs between the four selected surveys, and the smaller sample size in 2011 (due to financial constraints and economic problems), samples were representative of the Iranian adult population in all four surveys. Generally, a representative sample of urban and rural individuals was selected based on a multistage random cluster sampling method. The national postal code database, which includes addresses of all residential buildings in the country, was used as the sampling frame. Through systematic proportional to size cluster sampling, samples from both rural and urban areas were selected within each province of Iran. Blood samples were collected from all participants after fasting for at least 10 hours overnight. All blood samples were shipped on dry ice to the central laboratory located in Tehran for analysis.

### Definitions

To calculate the prevalence of diabetes, all participants aged 25 to 65 years were considered. However, to calculate the percentage of treatment received and the percentage of diabetes controlled only individuals identified as diabetic patients were included in the analysis. Indicators were stratified by age (25-39, 40-54, 55-65 years old), gender (female/male), place of residence (rural/urban), household economic status (first to fifth wealth quintile), and insurance health coverage. It should be mentioned that the information on households’ economic status and information on insurance coverage were collected just in two rounds. In addition, just in one round (2016) the glucose level was measured by two clinical markers of diabetes status, Hemoglobin A1C (HbA1c) and fasting blood sugar (FBS).

The criteria for diagnosing diabetes in the present study are as follows:

- Diabetes was defined as the presence of one of the following conditions: 1) FBS higher than 126 mg/dL or; 2) HbA1c of greater than or equal to 6.4% mmol/L or; 3) or self-report of the previous diagnosis of diabetes by medical professionals or taking medicine at the time of survey (oral glycemic medications in the last two weeks, insulin in the last two weeks) at the time of the survey. The questionnaire did not specify the type of diabetes (1 or 2).
- The awareness of diabetes was defined as those who self-reported physician-diagnosed diabetes among all participants with diabetes.
- The treatment of diabetes was defined as the percentage of diabetic patients who had taken diabetic medication regularly.
- The control of diabetes was defined as the percentage of diabetic patients whose FBS level was less than 140 mg/dL or their HbA1c level was under 7.0% mmol/L [19].

### Data analysis

Descriptive statistics (means and standard deviations for continuous variables, counts and percentage for categorical variables) were used to provide information on the prevalence of diabetes, diabetes awareness, treatment and control, stratified by age, sex, place of residence, wealth quintile, and health insurance status. Crude prevalence estimates of awareness, treatment, and control of diabetes were calculated and presented with 95% confidence interval (CI). Multivariate logistic regression analysis was used to identify factors (age, sex, place of residence, wealth quintile, health insurance status) associated with diabetes prevalence, awareness, treatment and control. Statistical significance was defined as a *P*-value< 0.05. Sample weights were incorporated into all analyses to provide generalizable estimates. All the statistical analyses were conducted with STATA version 13 software. Analyzes were not standardized by age and sex.

## Results

Table 1 summarizes the demographic characteristics of the participants in each round. The overall sample sizes of four surveys were: 2004 (*n* = 89,404), 2007 (*n* = 29,991), 2011 (*n* = 12,103), and 2016 (*n* = 30,541) of which we included individuals aged 25-65 years in the study as follows: 2004 (*n* = 70,961), 2007 (*n* = 23,942), 2011 (*n* = 7953), and 2016 (*n* = 23,734).

**Table 1:** General characteristics of the participants in four surveys

Variables	2004	2007	2011	2016
Total number of participants	89,404	29,991	12,103	30,541
Total number of participants aged 25-65	70,961	23,942	7953	23,734
Total number of participants with biochemical assessments aged 25-65	70,450	20,860	7641	16,758
Gender				
Female	35,138	11,972	4456	12,473
Male	35,813	11,970	3497	11,301
Age category (year)				
25-39	25,955	8238	3095	10,766
40-54	27,656	9807	2209	8589
55-65	17,350	5897	2649	4379
Health insurance				
Yes	–	23,868	–	23,233
No	–	74	–	501
Supplementary health insurance coverage (%)	–	–	–	21.1 (20.7-21.6)
Fasting blood sugar average	94.9 (91.4-98.3)	89.2 (88.6-89.8)	93.0 (91.5-94.5)	99.5 (98.8-100.1)
Over weight prevalence (%)	28.6	46.0 (45-47)	48.2 (45.5-82.7)	59.3 (58.7-59.9)
Obesity prevalence (%)	10.8	16.7 (16-17.6)	16.8 (15.2-18.35)	22.7 (22.2-23.2)
Mean BMI (kg/m2)	24.8 (24.6-24.9)	25.1)25-25.2(	25.3 (25.1-25.6)	26.5 (26.5-26.6)

*BMI* Body Mass Index

*Data on supplementary health insurance was collected only in 2016. Data on basic health insurance were not collected in 2004 and 2011

The prevalence of diabetes among 25-65 years old adults during 2004-2016 by gender, age, place of residence, wealth quintile and insurance coverage are presented in Table 2. The overall prevalence of diabetes based on FBS increased from 8.4 to 13.2% during the period investigated. In 2016, the prevalence based on HBA1c was 13.5%, which is 0.3% higher than the estimated prevalence based on FBS. The prevalence increased with advancing age in all years. Diabetes was more prevalent among the age group of 55 to 65 years (26.8% in 2016). The prevalence of diabetes increased among both sexes in the period with a higher absolute increase among females (5.4% vs. 3.9%). In the study period, on average, diabetes was more prevalent in urban areas compared to rural areas (11.7% vs. 8%). Diabetes was more prevalent among individuals with health insurance compared to those uninsured. The highest prevalence of diabetes was among the third quintile in 2016 (15.3%) and the lowest one was for the first quintile (10.7%).

**Table 2:** Prevalence of diabetes among Iranian population aged 25-65 in four rounds

Variables	2004%(95%CI)	2007%(95%CI)	2011%(95%CI)	2016%(95%CI) Based on HbA1c	2016%(95%CI) Based on FBS
Total	8.4 (8.2-8.7)	9 (8.5-9.6)	11.1 (10-12.1)	13.5 (12.9-14.2)	13.2 (12.6-13.8)
Gender					
Female	9.2 (8.9-10.5)	9.7 (8.9-105)	12.5 (11.8-14)	14.8 (13.9-15.6)	14.6 (13.7-15.5)
Male	7.6 (7.23-8)	8.3 (7.5-9.1)	9.7 (7.8-10.9)	12 (11.2-12.9)	11.5 (10.6-12.3)
Age category (year)					
25-39	3.8 (3.5-4.1)	4.2 (3.5-4.9)	5 (3.6-6.3)	5.3 (4.7-5.9)	5.3 (4.7-5.9)
40-54	11.3 (10.8-11.8)	11.9 (10.9-13)	14.6 (12.6-16.6)	14.4 (13.3-15.4)	14 (13-15.1)
55-65	17.4 (16.6-18.2)	18.5 (17-20.1)	24.4 (22-26.9)	27.8 (26-29.5)	26.8 (25-28.6)
Place of residence					
Urban	9.8 (9.4-10.2)	10.1 (9.3-10.9)	12.5 (11.1-13.9)	14.7 (13.9-15.5)	14.3 (13.5-15.1)
Rural	6.2 (5.8-7)	7.3 (6.5-8.1)	8 (6.6-9.4)	10.8 (9.9-11.6)	10.5 (9.6-11.3)
Wealth quintile					
First quintile	–	–	7.4 (5.8-8.9)	11.2 (10-12.5)	10.7 (9.5-11.9)
Second quintile	–	–	11 (8.8-13.2)	13.7 (12.7-15.1)	13.8 (12.4-15.3)
Third quintile	–	–	14 (11.2-16.7)	15.7 (14.2-17.3)	15.3 (13.7-16.8)
Fourth quintile	–	–	11.4 (9.4-13.5)	13.9 (12.5-15.2)	13.4 (12-14.7)
Fifth quintile	–	–	11.6 (8.5-14.8)	13.4 (12-14.8)	13.3 (11.9-14.7)
Health insurance					
Yes	–	9.6 (8.9-10.2)	–	13.9 (13.2-14.5)	13.6 (13-14.2)
No	–	6.9 (5.8-8)	–	8.9 (6.8-11)	8.3 (6.2-10.3)

* Data on wealth quintiles were collected only in two surveys: 2011, 2016. Data on supplementary health insurance was collected only in 2016 survey. Data on basic health insurance were collected in 2007 and 2016 surveys

As Table 3 illustrates, the overall proportion of patients who were aware of their diabetes increased from 53.5 to 82.2% over the period 2004-2016. The awareness increased among all age groups, which increased with advancing age. The age group of 55-65 years in 2004, 2007 and 2011 had the highest awareness of the disease (61.1, 77.6, and 75.9%), however; in 2016 the age group of 25-39 years had the highest level of awareness (84.3%). The level of awareness among males and females increased during the study period, with a higher level of awareness among females in 2016 (84.9% vs. 78%). However, the absolute growth was greater among men compared to women during the study years (30.9%vs. 26.5%). Self-awareness of diabetes also increased in rural and urban areas in the study period. Overall, the absolute increase was higher in rural areas compared to that in urban areas (33.7% vs. 27.2%). Diabetes awareness was higher among participants with health insurance compared to those without health insurance with an upward trend in both groups over time. Diabetes awareness increased among all five wealth quintiles during the study period. The third quintile had the highest awareness (86.3%) in 2016 and the lowest awareness was observed among the first quintile (80.9%).

**Table 3:** Awareness, treatment and control of diabetes among diabetic patients aged 25-65 in four rounds

Variables		2004%(95%CI)	2007%(95%CI)	2011%(95%CI)	2016%(95%CI) Based on HbA1c	2016%(95%CI) Based on FBS
Total						
awareness		53.5 (51.8-55.2)	65.6 (62.3-68.8)	70.5 (65.4-75.6)	79.1 (77.2-81.1)	82.2 (80.3-84.1)
treatment		35.9 (34.3-37.5)	42 (37.8-45.3)	46 (41.1-50.9)	38 (35.6-40.4)	39.6 (37.1-42.1)
control		14.5 (13.4-15.6)	20.8 (18.2-23.5)	20.4 (16.5-24.3)	14.3 (12.4-16.2)	18.5 (16.5-20.4)
Gender						
Female	awareness	58.4 (56.2-60.7)	71.8 (67.7-76)	74.1 (67.4-80.7)	82.6 (80.3-85)	84.9 (82.6-87.1)
	treatment	39.8 (37.7-41.9)	46.4 (42.1-50.6)	49.9 (43.7-56.1)	38.5 (35.3-41.7)	40.1 (36.9-43.4)
	control	16.4 (14.8-18)	21.6 (18.3-24.9)	19.5 (15.2-23.9)	14.6 (12-17.1)	18.8 (16.2-21.4)
Male	awareness	47.1 (44.4-49. 7)	57.7 (52.6-62.8)	65.2 (57.1-73.2)	73.8 (70.6-77.1)	78 (74.6-81.3)
	treatment	30.8 (28.5-33.1)	36.6 (31.5-41.6)	40.1 (32-48.1)	37.3 (33.6-41.1)	38.8 (34.9-42.7)
	control	12.1 (10.5-13.7)	19.9 (15.5-24.2)	21.8 (14.6-29)	13.9 (11.2-16.6)	17.9 (15-20.9)
Age category (year)						
25-39	awareness	40.3 (36-44.6)	48.6 (39.7-57.7)	55.9 (40.9-70.3)	83.4 (79.4-87.5)	84.3 (80-88.5)
	treatment	21.5 (18-25)	20.6 (13.2-28)	19.9 (10.4-29.5)	14.8 (10.4-19.1)	14.8 (10.4-19.2)
	control	11.4 (8.7-14.2)	13.7 (7-20.3)	11 (3.8-18.3)	9 (5.4-12.6)	9.6 (6-13.3)
40-54	awareness	54.9 (52.5-57.4)	66.2 (61.8-70.6)	74.4 (67.8-80.9)	77.5 (74.4-80.6)	80.6 (77.6-83.6)
	treatment	36.8 (34.5-39.2)	43.4 (38.7-48.1)	51.8 (44.6-59)	34.6 (30.7-38.4)	36.2 (32.2-40.1)
	control	13.9 (12.2-15.5)	20.2 (16.5-23.8)	22.6 (16.1-29.1)	11.9 (9.2-14.6)	15 (12.4-17.6)
55-65	awareness	61.1 (58.8-63.5)	77.6 (74-81.1)	75.9 (70.9-80.9)	79.1 (76.1-82.1)	82.9 (79.9-85.9)
	treatment	45.2 (42.8-47.6)	56.2 (51.7-60.7)	56.8 (51-62.6)	49.8 (46-53.5)	52.1 (48.3-56)
	control	17.7 (15.9-19.5)	27.3 (22.9-31.6)	24.2 (19.5-28.9)	18.5 (15.3-21.8)	25 (21.9-28.5)
Place of residence						
Urban	awareness	55.5 (53.5-57.5)	64.8 (60.8-68.7)	70.5 (64.4-76.7)	79.3 (77.1-81.6)	82.7 (80-84.5)
	treatment	38.1 (36.2-40)	42.4 (38.4-46.4)	47.3 (41.4-53.2)	38.1 (35.2-41.1)	39.3 (36.3-42.3)
	control	14.7 (13.4-16.1)	20.6 (17.5-23.7)	21.4 (16.7-26)	14.1 (11.8-16.4)	18.5 (16.2-20.9)
Rural	awareness	48.2 (45 -51.4)	67.3 (61.8-72.8)	70.6 (62.2-79.1)	78.5 (75-82)	81.9 (78.6-85.3)
	treatment	30 (27.2-32.8)	41.1 (35.6-46.7)	41.9 (33.3-50. 6)	37.7 (33.7-41.7)	40.5 (36.4-44.7)
	control	13.9 (11.8-16)	21.2 (16.2-26.3)	17.4 (11.2-23.6)	15 (12.1-18)	18.3 (15.1-21.6)
Wealth quintile						
First quintile	awareness	–	–	78.6 (70.1-87.1)	76.1 (71.1-81.1)	80.9 (76.2-85.7)
	treatment	–	–	45.7 (34.6-56.9)	34.6 (28.9-40.3)	37.3 (31.4-43.2)
	control	–	–	22.9 (13.1-32.7)	15.6 (11.7-19.5)	19.8 (15.4-24.2)
Second quintile	awareness	–	–	66.7 (56.8-76.6)	80.9 (76.5-84.67)	82.1 (77.9-86.9)
	treatment	–	–	43.8 (34.1-53.6)	43 (37.2-48.8)	43.6 (37.8-49.4)
	control	–	–	21.1 (13.7-28.6)	13.8 (10.1-17.5)	17.6 (13.4-21.8)
Third quintile	awareness	–	–	70.3 (58.5-82.2)	82.9 (78.8-86.1)	86.3 (83-89.5)
	treatment	–	–	48 (37.2-58.8)	38.1 (32.8-43.3)	40.2 (34.8-45.6)
	control	–	–	24.4 (15.4-33.5)	14.83 (10.4-19.3)	19.7 (14.6-23.9)
Fourth quintile	awareness	–	–	70.5 (61.1-79.9)	78.5 (74.1-82.8)	82.3 (77.9-86.6)
	treatment	–	–	45.8 (36.5-55.1)	39.4 (34.2-44.6)	41.6 (36.1-47)
	control	–	–	15.6 (9.4-21.8)	14 (9.8-18.2)	20.4 (15.7-25.2)
Fifth quintile	awareness	–	–	69 (54.6-83.4)	82.1 (78.1-86.1)	83.2 (79-87.4)
	treatment	–	–	46.9 (33-60.9)	36.2 (30.8-41.7)	36.4 (30.8-42)
	control	–	–	16.6 (7.4-25.9)	14.4 (9.8-19)	16.9 (13-20.8)
Health insurance						
Yes	awareness	–	66.5 (63-70)	–	80.4 (78.5-82.3)	93.2 (84.1-81.8)
	treatment	–	43.8 (40.2-47.3)	–	38.4 (35.9-40.9)	39.9 (37.3-42.4)
	control	–	21.9 (19-24.8)	–	14.3 (12.4-16.3)	20.1 (10.4-29.9)
No	awareness	–	60.8 (52.4-69.2)	–	73.4 (62.8-84)	80.5 (69.7-91.3)
	treatment	–	32.9 (25.1-40.6)	–	38.5 (26.6-50.3)	41.8 (29.1-54.5)
	control	–	15.1 (9.1-21)	–	15.2 (6.8-23.7)	18.5 (16.5-20.6)

*Data on wealth quintiles were collected only in two surveys: 2011, 2016. Data on supplementary health insurance was collected only in 2016 survey. Data on basic health insurance were collected in 2007 and 2016 surveys

Table 3 also shows the proportion of diabetes treatment and control during the investigated period among the diabetic patient population. The proportion of patients received treatment was 35.9% in 2004 and increased to 46% in 2011 but then decreased to 39.6% in 2016. The proportion of diabetic patients were on treatment increased with advancing age, according to which the age group of 65-55 years had the highest level of receiving treatment in 2016 (52.1%). A higher percentage of diabetic women received treatment for the condition compared to men in all investigated years. The proportion of diabetic patients on treatment in rural and urban areas increased from 2004 to 2011 and then declined in 2016. The proportion of patients received treatment in urban and rural areas were 38.1 and 30% in 2004, 47.3 and 41.9% in 2011 and 39.3 and 40.5% in 2016, respectively.

**Fig. 1:**
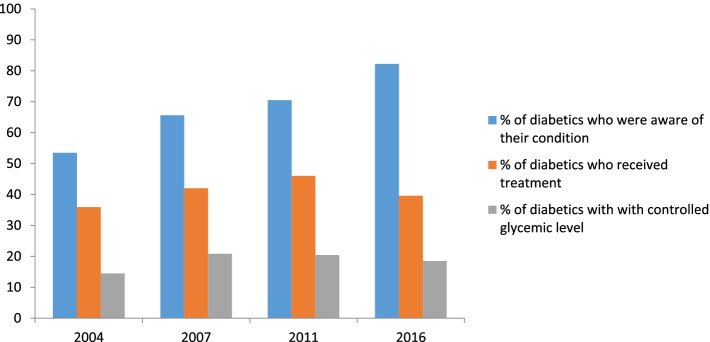
Percentage of diabetes awareness, treatment and control among diabetic patients. The bar chart demonstrating % of patients who were aware of their condition, who receive treatment and who had controlled blood glucose level in 2004, 2007, 2011 and 2016 surveys (based on FBS test)

Diabetic patients had better glycemic control in 2016 compared to 2004 (18.5% vs. 14.5%), with an absolute increase by 4% (Table 3). The proportion of diabetic patients with controlled FBS increased with advancing age in all years of the study. The proportion of diabetic males and females with controlled blood glucose level increased from 12.1 and 16.4% in 2004 to 17.9 and 18.8% in 2016, respectively. It was higher among females in three rounds (2004, 2007 and 2016) and in one round (2011) it was higher among males (21.8% vs. 19. 5%). The proportion of individuals with controlled diabetes in urban areas increased from 2004 to 2011 and then decreased to 18.5% in 2016, but overall, it increased during the period 2004-2016 by 3.8%. In rural areas, it increased from 13.9% in 2004 to 21.2% in 2007 and then decreased to 18.3% in 2016. Percentage of controlled diabetes was higher among diabetics with health insurance than those without health insurance in investigated years (21.9% vs. 15.1% in 2007 and 20.1% vs. 18.5% in 2016). The proportion of controlled diabetes in the first, second and third wealth quintiles decreased in 2016 compared to 2011 and increased among the fourth and fifth quintiles during the period. The highest and the lowest proportions of controlled diabetes were observed among the fourth quintile (20.4%) and fifth quintile (16.9%) in 2016. Figure 1 illustrates the proportion of diabetic patients who were aware of their condition, who were on treatment and who had adequate glycemic control in four rounds of the study.

Logistic regression was used to identify socio-demographic factors associated with the levels of awareness, treatment and control of diabetes mellitus. The initial model included variables for gender, age, place of residence, wealth quintile, and health insurance. Factors for which *P* was greater than or equal to 0.05 were removed from the final model. The results are summarized in Tables 4, 5, 6 and 7.

**Table 4:** Multivariate logistic regression analyses on risk factors for diabetes among Iranian aged 25-65 in 2016

Variables	Based on FBS		Based on HbA1C	
	OR (95%CI)	*p*-value	OR (95%CI)	*p*-value
Gender				
Female	1	Ref	1	Ref
Male	0.74 (0.66-0.83)	< 0.001	0.77 (0.69-0.86)	< 0.001
Age category (year)				
25-39	1	Ref	1	Ref
40-54	2.84 (2.44-3.31)	< 0.001	2.88 (2.48-3.34)	< 0.001
55-65	6.23 (5.32-7.30)	< 0.001	6.43 (5.51-7.50)	< 0.001
Place of residence				
Rural	1	Ref	1	Ref
Urban	1.31 (1.14-1.51)	< 0.001	1.33 (1.16-1.53)	< 0.001
Wealth quintile				
First quintile	1	Ref	1	Ref
Second	1.10 (0.91-1.33)	0.284	1.03 (0.85-1.24)	0.74
Third	1.27 (1.05-1.54)	0.013	1.23 (1.02-1.48)	0.03
Fourth	1.06 (0.87-1.30)	0.505	1.04 (0.85-1.26)	0.671
Fifth	0.97 (0.79-1.21)	0.853	0.91 (0.74-1.13)	0.414
Health insurance				
No	1	Ref	1	Ref
Yes	1.41 (1.06-1.87)	0.018	1.35 (1.02-1.78)	0.03
Supplementary health insurance				
No	1	Ref	1	Ref
Yes	1.16 (1.01-1.35)	0.023	1.18 (1.02-1.36)	0.019

*Ref* Reference category, *CI* Confidence interval

*All variables in the table included in the final model

**Table 5:** Multivariate logistic regression analyses on risk factors for diabetes awareness among diabetic patients aged 25-65 in 2016

Variables	Based on FBS		Based on HbA1C	
	OR (95%CI)	*p*-value	OR (95%CI)	*p*-value
Gender				
Female	1	Ref	1	Ref
Male	0.58 (0.45-0.77)	< 0.001	0.53 (0.42-0.68)	< 0.001
Age category (year)				
25-39	1	Ref	1	Ref
40-54	0.75 (0.51-1.09)	0.139	0.64 (0.45-0.91)	0.013
55-65	0.88 (0.60-1.30)	0.545	0.75 (0.53-1.08)	0.128
Place of residence				
Rural	Not significant in initial model			
Urban				
Wealth quintile				
First quintile	Not significant in initial model		1	Ref
Second			1.2 (0.82-1.78)	0.338
Third			1.4 (0.95-2.05)	0.086
Fourth			1.1 (0.74-1.62)	0.62
Fifth			1.42 (0.94-2.14)	0.091
Health insurance				
No	Not significant in initial model			
Yes				
Supplementary health insurance				
No	1	Ref	1	Ref
Yes	1.56 (1.14-2.14)	0.005	1.45 (1.08-1.94)	0.013

*Ref* Reference category, *CI* Confidence Interval

* Based on FBS, variables: place of residence, wealth quintile and health insurance were not significant in the initial model and were not entered into the final model. Based on HbA1C, variables: place of residence and health insurance were not significant in the initial model and were not entered into the final model

**Table 6:** Multivariate logistic regression analyses on risk factors for diabetes treatment among diabetic patients aged 25-65 in 2016

Variables	Based on FBS		Based on HbA1C	
	OR (95%CI)	*p*-value	OR (95%CI)	*p*-value
Gender				
Female	1	Ref	1	Ref
Male	0.88 (0.71-1.11)	0.303	0.92 (0.73-1.14)	0.466
Age category (year)				
25-39	1	Ref	1	Ref
40-54	3.34 (2.25-4.96)	< 0.001	3.07 (2.07-4.54)	< 0.001
55-65	6.49 (4.39-9.60)	< 0.001	5.84 (3.97-8.59)	< 0.001
Place of residence				
Rural	Not significant in initial model			
Urban				
Wealth quintile				
First quintile	Not significant in Initial model		1	Ref
Second			1.34 (0.93-1.91)	0.107
Third			1.12 (0.79-1.58)	0.507
Fourth			1.17 (0.83-1.66)	0.36
Fifth			1.09 (0.74-1.61)	0.645
Health insurance				
No	Not significant in initial model			
Yes				
Supplementary health insurance				
No	1	Ref	1	Ref
Yes	1.06 (0.83-1.36)	0.574	1.10 (0.84-1.42)	0.462

*Ref* Reference category, *CI* Confidence Interval

*Based on FBS, variables: place of residence, wealth quintile and health insurance were not significant in the initial model and were not entered into the final model. Based on HbA1C, variables: place of residence and health insurance were not significant in the initial model and were not entered into the final model

**Table 7:** Multivariate logistic regression analyses on risk factors for diabetes control among diabetic patients aged 25-65 in 2016

Variables	Based on FBS		Based on HbA1C	
	OR (95%CI)	*p*-value	OR (95%CI)	*p*-value
Gender				
Female	1	Ref	1	Ref
Male	0.90 (0.69-1.18)	0.468	0.91 (0.67-1.25)	0.596
Age category (year)				
25-39	1	Ref	1	Ref
40-54	1.70 (1.06-2.73)	0.028	1.36 (0.82-2.26)	0.228
55-65	3.19 (1.99-5.10)	< 0.001	2.30 (1.41-3.76)	< 0.001
Place of residence				
Rural	Not significant in initial model			
Urban				
Wealth quintile				
First quintile	Not significant in initial model			
Second				
Third				
Fourth				
Fifth				
Health insurance				
No	Not significant in initial model			
Yes				
Supplementary health insurance				
No	1	Ref	Not significant in initial model	
Yes	1.17 (0.87-1.56)	0.278		

*Ref* Reference category, *CI* Confidence interval

*Based on FBS, variables: place of residence, wealth quintile and health insurance were not significant in the initial model and were not entered into the final model. Based on HbA1C, variables: place of residence, wealth quintile, health insurance and supplementary health insurance were not significant in the initial model and were not entered into the final model

Table 4 presents the odds ratios for covariates and diabetes prevalence based on FBS and HbA1C in 2016. Male participants were less likely to have diabetes compared to females (based on FBS: OR = 0.74, CI: 0.66 to 0.83; based on HbA1c: OR = 0.77, CI: 0.69 to 0.86). Both age groups of 40-54 (based on FBS: OR = 2.84, CI: 2.44 to 3.31; based on HbA1c: OR = 2.88, CI: 2.48 to 3.34) and 55-65 years (based on FBS: OR = 6.23, CI: 5.32 to 7.3; based on HbA1c: OR = 6.43, CI: 5.51 to 7.5) had a greater probability of having diabetes than the 25-39 age group. Individuals residing in urban areas were more likely to have diabetes compared to those living in rural areas (based on FBS: OR = 1.31, CI: 1.14 to 1.51; based on HbA1c: OR = 1.33, CI: 1.16 to 1.53). Individuals in the third quintile were more likely to have diabetes compared to other wealth quintiles (based on FBS: OR = 1.27, CI: 1.05 to 1.54; based on HbA1c: OR = 1.23, CI: 1.02 to 1.48). Insured participants were more likely to have diabetes compared to uninsured individuals for both basic (based on FBS: OR=1.41, CI: 1.06 to 1.87; based on HbA1c: OR=1.35, CI: 1.02 to 1.78) and supplementary health insurance (based on FBS: OR = 1.16, CI: 1.01 to 1.35; based on HbA1c: OR = 1.18, CI: 1.02 to 1.36).

Table 5 shows the odds ratios for covariates and diabetes awareness based of FBS and HbA1C in 2016. Male participants were less likely to be aware of the disease (based on FBS: OR = 0.58, CI: 0.45 to 0.77; based on HbA1c: OR = 0.53, CI: 0.42 to 0.68). Individuals covered by supplementary health insurance were more likely to be aware of their diabetic condition (based on FBS: OR = 1.56, CI: 1.14 to 2.14; based on HbA1c: OR = 1.45, CI: 1.08 to 1.94). The age group 40-54 years was more likely to be aware of their hyperglycemic condition based on HbA1c (OR = 0.64, CI: 0.45 to 0.91).

As shown in Table 6, the relationship between receiving treatment and age groups of 40-54 (based on FBS: OR = 3.34, CI: 2.25 to 4.96; based on HbA1c: OR = 3.07, CI: 2.07 to 4.54) and 55-65 years (based on FBS: OR = 6.49, CI: 4.39 to 9.60; based on HbA1c: OR = 5.84, CI: 3.97 to 8.59) were significant in the final regression model. Second wealth quintile, the age groups of 40-54 and 55-65 years had higher odds of receiving treatment for diabetes in the initial model. The odds of receiving treatment were higher among the age groups of 40-54 and 55-65 years in the final model too.

In the initial regression model, the age groups of 40-54 and 55-65 years and individuals with supplementary health insurance were more likely to have controlled diabetes based on FBS. As Table 7 shows, in the final model, two age groups of 40-54 (based on FBS: OR = 1.70, CI 1.06 to 2.73) and 55-65 years (based on FBS: OR = 3.19, CI 1.99 to 5.10; based on HbA1c: OR = 2.3, CI 1.41 to 3.76) were more likely to have controlled glucose levels.

## Discussion

Diabetes is a complex health problem that results in significant morbidity and mortality and health care resource utilization, particularly in developing countries such as Iran [3, 8, 20]. Using nationally representative data, this study analyzed the prevalence, awareness, treatment and glycemic control of diabetes for a period of 12 years among Iranians aged 25-65 years old. Our findings indicated that the prevalence of diabetes and diabetes awareness increased over time while diabetes treatment and glycemic control, despite growth, are not at an appropriate level yet.

Our findings revealed that the prevalence of diabetes has increased in Iran in both urban and rural areas in recent years. The prevalence of diabetes among individuals aged 25-65 was 8.4% in 2004 and increased to 13.2% in 2016, which was higher than the worldwide average projected by International Diabetes Federation for 2017(8.4%) [21]. Similar to other countries located in the MENA region, Iran has witness an increasing trend in recent years, contributed by different factors including genetics, obesity, physical inactivity, urbanization, and poor nutritional habits [22, 23]. In line with the present study, the prevalence of diabetes in most similar national studies was reported to be increasing, ranging from 7.4 to 24.5% [24–27]. These differences in the figures reported for diabetes prevalence can be attributed to differences associated with methods including different age distribution, different sample sizes, different time period, different regional focus, as well as lack of uniform diagnostic criteria. However, it should be noted that the upward trend in diabetes prevalence in Iran has been reported regardless of whether the prevalence is measured using self-reported data or a clinical marker of diabetes status, such as FBS or HbA1c. Hence, due to the numerous complications associated with diabetes, it is necessary to pay serious attention to the issue in health policies.

Regression analyses showed that higher prevalence of diabetes was associated with older age, female sex and residing in the urban area. The results of similar studies suggest that risk for diabetes increases with age after 40 years [25, 28, 29]. Further, one of the reasons for the higher incidence of diabetes among women is the incidence of gestational diabetes [30], which reveals the importance of designing high quality of antenatal care to detect and manage gestational diabetes. Similar to several national and international studies, our results indicate a higher prevalence of diabetes in urban areas compared to rural areas [24, 31–33], which may be due to changes in lifestyles and health behaviors such as diet and physical activity. Although there was higher diabetes prevalence in urban areas, our study showed no considerable differences in diabetes awareness and control between rural and urban areas. These findings are not consistent with those of previous studies reported a better disease management in rural areas of Iran because of the expended primary health care systems with trained community health-care workers [34, 35].

The awareness of diabetes among diabetic patients was about 53.5% at the beginning of the study period (2004) that reached 82.2% at the end of the period (2016), which is higher than the estimated awareness (51%) in the MENA region in 2017 [36]. According to the International Diabetes Federation, one in two people with diabetes is undiagnosed worldwide [4]. Various studies have shown that the awareness level about diabetes has a significant relationship with glycemic control and reducing its complications [37, 38]. Several reasons can be put forward for the increased awareness. First, in our study, the diabetes prevalence has been rising during the study period and therefore, the awareness growth could be due to the increased prevalence. Second, increased health promotion activities within the healthcare system as well as social media campaigns to educate people about healthy lifestyle could be other reasons. Finally, screening services for diabetes provided by PHC facilities may have a role in raising awareness. The logistic regression results indicate that diabetes awareness increases with age, which is consistent with the results of other studies [39–41]. Women are more likely to be aware of their risks of diabetes and have adequate glycemic control compared to men [33, 41], that was observed in our study as well.

In the present study, the treatment level increased from 2004 to 2011 and then decreased to 39.6% in 2016 whereas the trend was expected to be upward due to the increased awareness. In the present study, the proportion of diabetic patients with controlled FBS increased from 2004 to 2011 and then decreased to 18.5% in 2016. Overall, the percentage of diabetic patients with controlled diabetes increased by almost 4% during the study period. However, the percentage of patients received treatment and the percentage of patients with adequate glycemic control were expected to be much higher, indicating that the management of diabetes is suboptimal in Iran far from the targeted goals [14, 42]. Several studies also challenged the effectiveness of the prevention and treatment programs to control diabetes and its complications in Iran at the system level [43, 44].

Our results indicated that disease awareness and control were higher among patients with health insurance than those without insurance. Since patients with health insurance have greater access to health services than those without insurance, in most national and international studies including our study, people with health insurance have been found to be more likely to be diagnosed with diabetes than those without insurance [26, 45, 46]. A number of studies have reported that the uninsured are much less likely to receive routine checkups or preventive services [47, 48], tend to be more severely ill when diagnosed, and receive less therapeutic care [49]. Diabetes has been recognized as a costly chronic condition that imposes a considerable cost on patients, especially lower-income patients. Therefore, to increase the level of diabetes control, it is necessary to provide financial support, such as strengthening insurance coverage and reducing copayments, particularly those of medicines, for low-income people to improve the financial access of these people to healthcare services. In recent years, due to the sanctions imposed by the US, there was a lack of access to essential medicine including insulin, because of either shortages or high prices. Previous studies have shown that although oral medication therapies for diabetic patients are affordable, insulin therapy is unaffordable for low-income patients in Iran, which was regarded as a significant barrier to treatment and reported as the major reason for forgone care [50, 51].

In our study, less than 25% of diabetic patients had adequate glycemic control. Despite the relatively high level of awareness, the level of control is still poor. The effective coverage of diabetes mellitus type 2 in people aged 15 years or above, is an indicator that represents progress in UHC for this disease [52]. Achieving this type of coverage requires a comprehensive health delivery system providing high quality services according to the needs of the people, leading to the improved health outcomes of the people receiving the interventions [53]. Further research is needed to identify the main drivers of the poor glycemic control among diabetic patients in Iran. However, what is evident is that redesigning the system of care for managing patients with diabetes to promote more accessible, affordable and good quality services is required.

This study had several limitations. First, the STEPS survey was unable to distinguish between type I and type II diabetes mellitus. Second, the single measurement of FBS, used in three rounds of the survey, may have overestimated the prevalence of diabetes either if participants were not truly fasting or may have underestimated it compared to an oral glucose tolerance test. For logistic and financial reasons, other markers for diabetes diagnosis (e.g. oral glucose tolerance test and HbA1c) were not measured. However, FBS is widely recognized as an acceptable screening test for diabetes and a good measure of diabetes control.

## Conclusion

The prevalence of diabetes in Iran is increasing and despite the relatively high awareness of the disease, receiving treatment and effective control of the disease are suboptimal. While several national policies to improve diabetes screening and care have been passed in recent years, it seems large gaps remain in disease detection and treatment. Further analysis of the reasons for low level of glycemic control among diabetic patients remains a subject of future research. Given the increasing trend of the diabetes prevalence, more population research is required to quantify the impact of diabetes in the future in Iran. It is suggested that more attention be paid to the comprehensive management of diabetes at the system level to reduce diabetes-related morbidity and mortality.

## Data Availability

The datasets analysed during the current study are not publicly available due to limitations imposed by  Iran's National Institute of Health Research (NIHR) data policy on data re-dissemination and use as the owner of the data, but are available from the corresponding author on reasonable request.

## Abbreviations

FBS: Fasting Blood Sugar
HbA1c: Hemoglobin A1C
HTP: Health Transformation Plan
MENA: Middle East and North Africa
MoHME: Ministry of Health and Medical Education
NCDs: Non-communicable Diseases
SDGs: Sustainable Development Goals
WHO: World Health Organization
